# Performance of universal and stratified computer-aided detection thresholds for chest x-ray-based tuberculosis screening: a cross-sectional, diagnostic accuracy study

**DOI:** 10.1016/j.landig.2025.100934

**Published:** 2025-11-26

**Authors:** Joowhan Sung, Peter James Kitonsa, Annet Nalutaaya, David Isooba, Susan Birabwa, Keneth Ndyabayunga, Rogers Okura, Jonathan Magezi, Deborah Nantale, Ivan Mugabi, Violet Nakiiza, David W Dowdy, Achilles Katamba, Emily A Kendall

**Affiliations:** Division of Infectious Diseases, Johns Hopkins University School of Medicine, Baltimore, MD, USA; Walimu, Uganda Tuberculosis Implementation Research Consortium, Kampala, Uganda; Walimu, Uganda Tuberculosis Implementation Research Consortium, Kampala, Uganda; Walimu, Uganda Tuberculosis Implementation Research Consortium, Kampala, Uganda; Walimu, Uganda Tuberculosis Implementation Research Consortium, Kampala, Uganda; Walimu, Uganda Tuberculosis Implementation Research Consortium, Kampala, Uganda; Walimu, Uganda Tuberculosis Implementation Research Consortium, Kampala, Uganda; Walimu, Uganda Tuberculosis Implementation Research Consortium, Kampala, Uganda; Walimu, Uganda Tuberculosis Implementation Research Consortium, Kampala, Uganda; Walimu, Uganda Tuberculosis Implementation Research Consortium, Kampala, Uganda; Walimu, Uganda Tuberculosis Implementation Research Consortium, Kampala, Uganda; Walimu, Uganda Tuberculosis Implementation Research Consortium, Kampala, Uganda; Department of Epidemiology, Johns Hopkins Bloomberg School of Public Health, Baltimore, MD, USA; Walimu, Uganda Tuberculosis Implementation Research Consortium, Kampala, Uganda; Makerere University College of Health Science, Department of Internal Medicine, Clinical Epidemiology and Biostatistics Unit, Kampala, Uganda; Division of Infectious Diseases, Johns Hopkins University School of Medicine, Baltimore, MD, USA; Walimu, Uganda Tuberculosis Implementation Research Consortium, Kampala, Uganda; Department of Epidemiology, Johns Hopkins Bloomberg School of Public Health, Baltimore, MD, USA

## Abstract

**Background:**

Computer-aided detection (CAD) software analyses chest x-rays for features suggestive of tuberculosis and provides a numeric abnormality score. However, estimates of CAD accuracy for tuberculosis screening are hindered by the scarcity of confirmatory data among people with lower x-ray scores, including those without symptoms. Additionally, the appropriate x-ray score thresholds for obtaining further testing might vary according to population and client characteristics. We aimed to evaluate the accuracy of CAD among all screened individuals and assess whether stratifying CAD thresholds by age and sex could improve performance.

**Methods:**

In this cross-sectional, diagnostic accuracy study, we screened for tuberculosis in individuals aged 15 years and older in Uganda using portable chest x-rays with CAD (qXR version 3.2). Participants not on active tuberculosis treatment were offered screening regardless of their symptoms. We included data from all participants from both facility-based and community-based sites who were screened from June 1, 2022 (study start), to March 31, 2024. Individuals with x-ray scores above a threshold of 0·1 (range 0–1) were asked to provide sputum for Xpert MTB/RIF Ultra (Xpert) testing. We estimated the diagnostic accuracy (sensitivity, specificity, and area under the curve [AUC]) of CAD for detecting Xpert-positive tuberculosis when using the same threshold for all individuals (under different assumptions about tuberculosis prevalence among people with x-ray scores <0·1), and compared this estimate with approaches stratified by age, sex, or both.

**Findings:**

54 840 individuals were assessed for eligibility, 52 835 of whom were screened for tuberculosis using CAD. The median age was 38 years (IQR 26–50), 23 586 (44·6%) participants were male, and 29 249 (55·4%) were female. 8949 (16·9%) had x-ray scores of 0·1 or more. Of 7219 participants with valid Xpert results, 382 (5·3%) were Xpert-positive, including 81 with trace results. Assuming 0·1% of participants with x-ray scores less than 0·1 would have been Xpert-positive if tested, qXR had an estimated AUC of 0·92 (95% CI 0·90–0·94) for Xpert-positive tuberculosis. Stratifying x-ray score thresholds according to age and sex improved accuracy; for example, at 96·1% (95% CI 95·9–96·3) specificity, estimated sensitivity was 75·0% (69·9–79·5) for a universal threshold (of ≥0·65) versus 76·9% (71·9–81·2) for thresholds stratified by age and sex (p=0·046).

**Interpretation:**

Our findings suggest that the accuracy of CAD for tuberculosis screening among all screening participants, including those without symptoms or abnormal chest x-rays, is higher than previously estimated. Stratifying x-ray score thresholds based on client characteristics such as age and sex could further improve accuracy, enabling a more effective and personalised approach to tuberculosis screening.

## Introduction

More than ten million people are estimated to develop tuberculosis each year, of whom more than 3 million are never reported to public health authorities.^1^ Improved case-finding strategies are urgently needed to reduce the global burden of tuberculosis.^2^ Chest x-ray is a useful tool for tuberculosis screening, with higher sensitivity than symptom-based screening and potential for high throughput at low cost.^3^ Computer-aided detection (CAD) systems, which use artificial intelligence (AI) to analyse chest x-rays, have recently emerged as a promising tool for scaling up chest x-ray-based tuberculosis screening.

An important point of uncertainty is the most appropriate threshold at which to refer screening participants for further evaluation. CAD products generate a score (x-ray score) that correlates with the probability of pulmonary tuberculosis. WHO recommends CAD calibration studies^4^ to determine the appropriate x-ray score threshold (CAD threshold) for each specific population and context. However, most existing studies have either evaluated the diagnostic accuracy of CAD among symptomatic individuals in clinical triage settings^5–10^ or offered sputum testing to people who are symptom negative only if they had tuberculosis-suggestive x-rays.^11–15^ As a result, few data exist on the accuracy of CAD—and the optimal CAD threshold for further evaluation—among people with mildly abnormal chest radiographs and no known symptoms. These data are important given the likely contribution of asymptomatic tuberculosis to transmission of *Mycobacterium tuberculosis* in communities.^16^

In developing optimal CAD thresholds for community-based screening, ancillary data, such as age and sex, might be particularly important to consider. Most existing evaluations of CAD for tuberculosis screening have used a single threshold for all participants,^17–20^ thereby ignoring known associations of tuberculosis risk with sex (ie, higher prevalence in men)^21,22^ and age (ie, higher probability of x-ray abnormalities representing non-tuberculosis conditions in older individuals).^23^ Therefore, tailoring CAD thresholds based on age and sex might improve performance. We therefore analysed results from an ongoing tuberculosis case-finding study in Uganda with the aim of evaluating the diagnostic accuracy of CAD among screening participants and assessing the effect of stratifying CAD thresholds according to participant demographics.

## Methods

### Study design and participants

In this cross-sectional, diagnostic accuracy study, we conducted community-based tuberculosis screening using portable digital chest x-ray with CAD as part of an ongoing cluster-randomised trial in Uganda (CHASE-TB, NCT05285202). Adults or adolescents aged 15 years and older and not on active tuberculosis treatment were eligible for the study, regardless of symptoms.

Participants were recruited to undergo screening in testing tents set up either near district-level health facilities (facility-based sites) or in areas with high traffic, such as transit hubs or markets, in neighbourhoods and villages believed to have a high tuberculosis prevalence (community-based sites), in peri-urban and rural areas surrounding Kampala. At facility-based sites, recruitment was not limited to patients seeking care at the facilities, but also included companions, staff, and passersby. At community-based sites, participants were recruited by interacting with anyone passing by, and by visiting nearby homes and shops when enrolment slowed. The present analysis retrospectively considers data from all participants from both facility-based and community-based sites who were screened from June 1, 2022 (study start), to March 31, 2024.

The study was approved by the institutional review boards at the Johns Hopkins University School of Medicine (Baltimore, MD, USA; IRB00300939) and Makerere University School of Public Health (Kampala, Uganda; SPH-2021-181). Oral informed consent (or assent with parental consent) was obtained from all study participants.

### Procedures

All consenting participants completed a standard questionnaire that collected demographic information (including self-report of age, sex [male or female], and race), smoking history (added 5 months after study initiation), known tuberculosis exposures, 30-day tuberculosis symptom history, tuberculosis treatment history, and HIV status. All participants who were not pregnant were offered screening with digital chest x-ray using a portable x-ray device. Chest radiographs were then read in real-time by CAD software (qXR version 3.2) independently of all clinical data. Participants whose x-rays were assigned qXR tuberculosis scores (x-ray scores; range 0–1) higher than the prevailing threshold were asked to provide expectorated sputum, which was sent for Xpert MTB/RIF Ultra (Xpert) testing at a local health facility (without accompanying clinical information or x-ray results). X-ray scores are intended to discriminate tuberculosis status but are not directly interpretable as probabilities. The CAD threshold for Xpert testing was initially set to 0·5 and was adjusted to 0·2 after 1 month and to 0·1 after an additional 4 months, reflecting the distribution of x-ray scores and desire to use available testing capacity for research purposes.

### Statistical analysis

Participant characteristics were summarised as median (IQR), or as percentages for categorial variables, and were compared across groups using *t* tests and *χ*^2^ tests.

We estimated the performance (sensitivity, specificity, and area under the curve [AUC]) of CAD for detecting Xpert-positive tuberculosis among individuals who were not pregnant and could provide an expectorated sputum sample. Because participants with x-ray scores less than 0·1 were not asked for sputum, our primary analysis assumed that 0·1% of participants with x-ray scores less than 0·1 would be Xpert-positive (an estimate supported by analysis of CAD data from a study of universal Xpert Ultra screening in Uganda; appendix pp 4–6), with sensitivity analyses assuming Xpert-positive proportions ranging from 0·05% (half of our primary assumption as the lower limit) to 0·3% (the estimated national prevalence of Xpert-positive tuberculosis in Uganda^24^). For participants with an x-ray score less than 0·1, we assumed that the proportion who would successfully provide a sputum sample was similar to that of participants with scores between 0·1 and 0·19 (appendix p 6), and we assigned sputum production and Xpert status randomly. We also examined the correlation between x-ray scores and semi-quantitative Xpert results among participants with Xpert-positive sputum using the Spearman correlation coefficient.

We derived CAD thresholds stratified by age, sex, or both, under the principle that, to maximise the effect of screening under constrained confirmatory testing capacity, Xpert tests should be offered with a similar minimum pretest probability in all participant subgroups. To derive these stratified CAD thresholds, we first fitted shape-constrained (monotonically increasing) generalised additive models^25^ for each subgroup, using Xpert result as the outcome and x-ray score as the explanatory variable. We then identified, for each age and sex subgroup, the score at which the prevalence of Xpert positivity was estimated to be closest to 2% (and separately, closest to 1%, as a sensitivity analysis), corresponding to a resource threshold of 50 (and 100) Xpert tests required to produce one positive result. We took these scores as the stratified CAD thresholds for each age and sex subgroup and we estimated the overall sensitivity and specificity of CAD when thresholds stratified by age, sex, or both were used. Then, to compare the performance of a universal strategy against stratified approaches, we identified the universal CAD threshold that would match the specificity of each set of stratified thresholds, representing a fixed capacity for confirmatory testing of people who do not have tuberculosis. We compared sensitivities between the universal and stratified approaches to estimate the potential sensitivity gains achievable by stratifying thresholds under constrained confirmatory testing resources. We considered trace-positive Xpert Ultra results as positive in our primary analyses,^26^ but we also performed sensitivity analyses considering them as negative. Our sample size was estimated to provide 80% power to detect a 3-percentage-point difference in sensitivity between universal and stratified thresholds, with a two-sided α of 0·05 (appendix p 3).

Because participants with x-ray scores between 0·1 and 0·49 were not asked for sputum during the first 5 months of the 22-month study, we limited CAD threshold selection and accuracy evaluation to participants enrolled after the CAD threshold was lowered to 0·1. We then conducted a sensitivity analysis that included data from participants screened before the threshold change, using bootstrapping to recreate screening populations of the same size and x-ray score distribution as the full study population but with complete Xpert information (appendix p 8). Outcomes were estimated using the same set of thresholds selected in the primary analysis based on the smaller dataset, along with corresponding uncertainty. Statistical significance was defined as two-sided p<0·05. Analyses were conducted using Stata version 16·1 and R version 4·3.2.

### Role of the funding source

The funder of the study had no role in the study design, data collection, data analysis, data interpretation, or writing of the report.

## Results

54 840 individuals were assessed for study eligibility; 77 were on tuberculosis treatment, 12 did not consent, 1374 were pregnant and offered sputum testing without x-ray, and 542 eligible individuals did not have an x-ray result documented. Therefore, 52 835 participants were screened for tuberculosis using AI-interpreted digital x-rays with valid x-ray scores, including 45 758 screened after the CAD threshold was lowered to 0·1. Of the 52 835 participants who were screened, the median age was 38 years (IQR 26–50), 23 586 (44·6%) were male, 29 249 (55·4%) were female, 3478 (6·6%) reported known HIV infection, 724 (1·4%) reported a history of tuberculosis, and 16 857 (31·9%) reported cough within the past 30 days (table 1).

**Figure 1:**
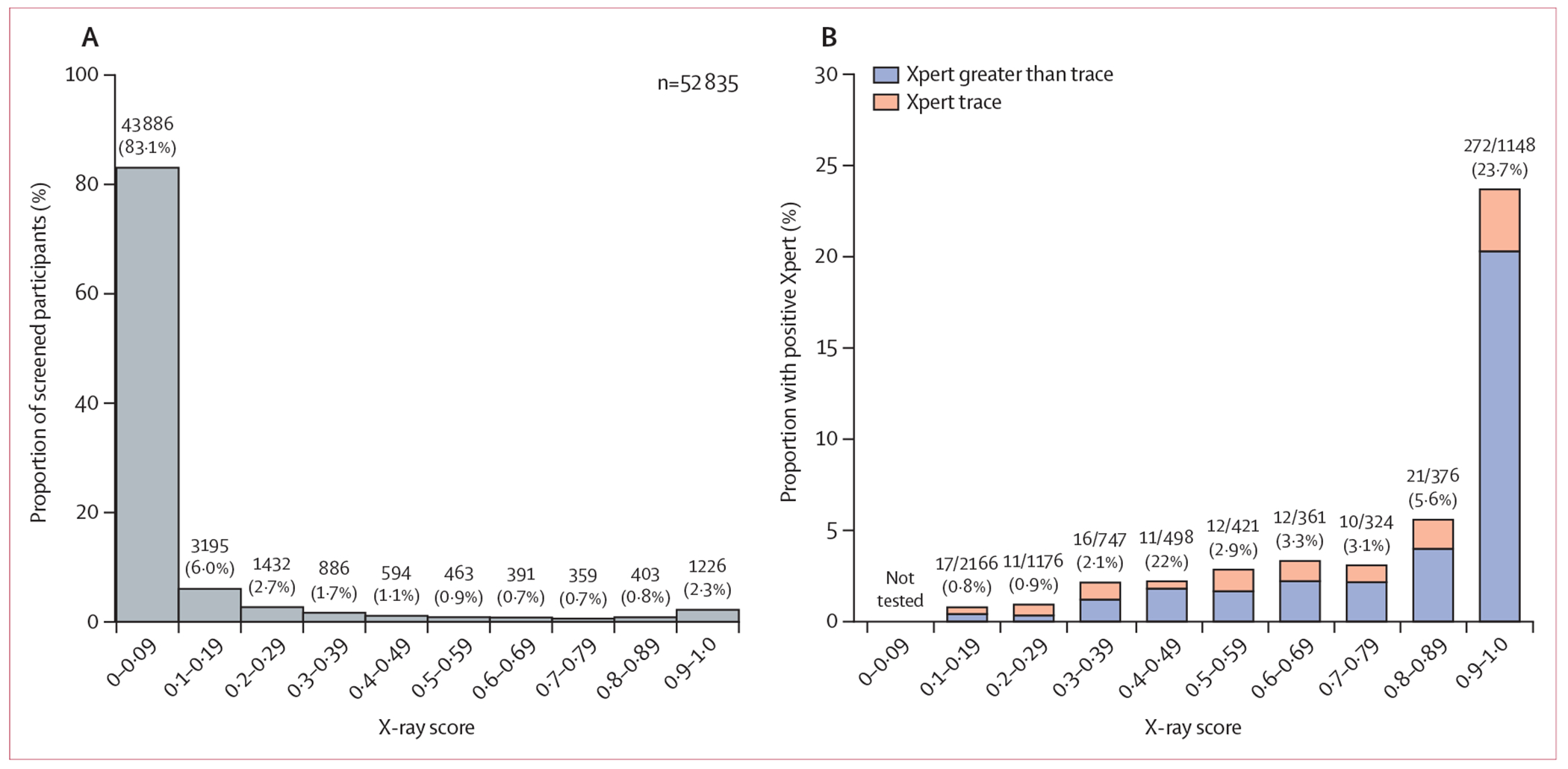
X-ray and Xpert results of community-screened adults (aged ≥15 years) in Uganda (A) The distribution of qXR tuberculosis scores among study participants who received x-ray-based tuberculosis screening in Uganda with computer-aided detection software. (B) The proportion of participants whose sputum Xpert results were positive within each x-ray score increment. qXR=computer-aided detection software qXR version 3.2. Xpert=Xpert MTB/RIF Ultra.

43 886 (83·1%) of 52 835 participants had an x-ray score less than 0·1, 6107 (11·6%) had a score between 0·1 and 0·49, 1616 (3·1%) had a score between 0·5 and 0·89, and 1226 (2·3%) had a score of 0·9 or more (figure 1A). 8038 (15·2%) participants were offered sputum testing, of whom 7239 (90·1%) provided sputum and 7219 (89·8%) had valid Xpert Ultra results. Of the valid Xpert results, 301 (4·2%) were positive at a level greater than trace, and an additional 81 (1·1%) were trace-positive. Younger age, male sex, tuberculosis symptoms, and previous tuberculosis treatment were associated with positive Xpert results (including trace; appendix p 9), including among individuals with x-ray scores between 0·1 and 0·5 (appendix p 10).

The relationship between x-ray scores and Xpert results is shown in figure 1B. Of 2166 participants with x-ray scores between 0·1 and 0·2, 17 (0·8%) had positive (including trace-positive) Xpert results. 23 (2·5%) of 919 participants with scores between 0·4 and 0·59 had positive Xpert results and 272 (23·7%) of 1148 with scores of 0·9 or higher had positive Xpert results (table 2). The proportion of study participants found to have Xpert-positive sputum was higher for men (289 [1·2%] of 23 586) than women (93 [0·3%] of 29 249), and was similar between age groups (189 [0·8%] of 24 607 aged 40 years and older *vs* 193 [0·7%] of 28 227 younger than 40 years). However, older participants had a higher prevalence of x-ray abnormalities detected by CAD than younger participants (x-ray score ≥0·1 in 6396 [26·0%] of 24 607 *vs* 2552 [9·0%] of 28 227]; appendix p 11).

45 758 (86·6%) of 52 835 participants were screened after the CAD threshold was lowered to 0·1. In these participants, the estimated x-ray scores corresponding to a 1% or 2% probability of Xpert positivity were: 0·11 or 0·47 for men aged 15–39 years, 0·25 or 0·53 for men aged 40 years and older, 0·28 or 0·52 for women aged 15–39 years, and 0·44 or 0·89 for women aged 40 years and older. Among all participants with positive Xpert results, semiquantitative Xpert results were weakly correlated with x-ray scores (Spearman’s correlation coefficient *r*=0·28; appendix p 12).

**Figure 2:**
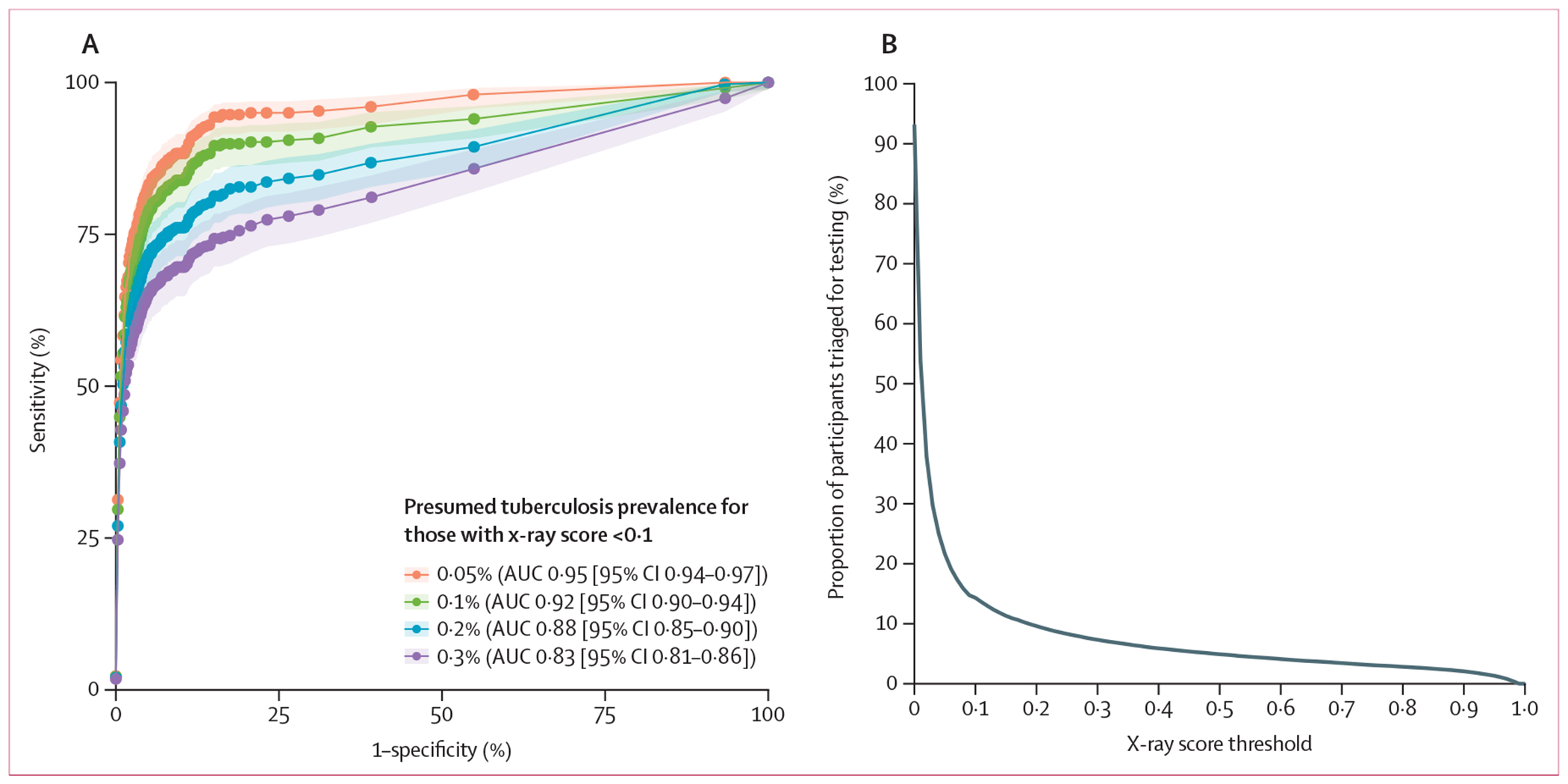
Diagnostic performance of CAD-based tuberculosis screening in Ugandan communities (A) ROC curves showing diagnostic performance of CAD software (qXR version 3.2) for sputum Xpert-positive tuberculosis. Uncertainty is shown as 95% CIs on the ROC curves and the AUC estimates. (B) The proportion of participants with x-ray scores greater than or equal to each x-ray score threshold thus qualifying for tuberculosis testing at that threshold. AUC=area under the curve. CAD=computer-aided detection. ROC=receiver operating characteristic. Xpert=Xpert MTB/RIF Ultra.

Assuming that 0·1% of people with x-ray scores less than 0·1 would test positive on sputum Xpert, community-based screening using CAD had an estimated AUC of 0·92 (95% CI 0·90–0·94) for Xpert-positive tuberculosis (figure 2). Under this assumption, the manufacturer-recommended universal threshold of 0·5 had an estimated sensitivity of 79·1% (95% CI 74·3–83·2) and specificity of 94·8% (94·6–95·0; appendix p 13). Lowering the threshold to 0·1 increased sensitivity to 89·9% (86·1–92·7) but decreased specificity to 83·6% (83·2–83·9). When we assumed a 0·3% prevalence of Xpert-positive tuberculosis among people with an x-ray score less than 0·1, the AUC decreased to 0·83 (0·81–0·86), sensitivity decreased to 65·6% (60·7–70·2) at a threshold of 0·5, or 74·5% (69·9–78·7) at a threshold of 0·1, and specificity remained similar (94·8% [94·6–95·0] at a threshold of 0·5 and 83·5 [83·2–83·9] at a threshold of 0·1).

In the sensitivity analysis incorporating data from participants enrolled early in the study, the AUC remained similar at 0·93 (0·91–0·94), assuming 0·1% prevalence among those with x-ray scores less than 0·1 (appendix p 14).

Compared with a universal threshold with matching specificity, thresholds that were stratified by both age and sex (at the scores corresponding to an estimated 2% Xpert positivity in each subgroup) had higher sensitivity (76·9% [95% CI 71·9–81·2] *vs* 75·0% [69·9–79·5]; p=0·046; table 3). The change in sensitivity from stratifying thresholds by age and sex was similar when stratified thresholds were set at the level corresponding to 1% Xpert positivity (85·1% [80·8–88·6] *vs* 83·5% [79·1–87·2]; p=0·18) or when higher or lower Xpert positivity were assumed for people with x-ray scores less than 0·1 (81·0% [76·2–85·0] *vs* 79·0% [74·0–83·2]; p=0·046 assuming 0·05% prevalence, or 63·8% [58·8–68·4] *vs* 62·2% [57·2–66·9]; p=0·046 assuming 0·3% prevalence). Thresholds stratified by both age and sex also resulted in higher sensitivities when trace-positive results were considered as negative (79·1% [73·8–83·6] *vs* 77·6% [72·1–82·2]; p=0·22) or when all eligible participants, including those who screened early in the study, were accounted for (73·4% [69·4–77·5] *vs* 72·4% [68·5–76·5]; p=0·21; appendix pp 15–18). Stratification by sex alone resulted in a smaller gain in sensitivity at higher thresholds (75·9% [70·9–80·3] *vs* 75·0% [69·9–79·5]; p=0·55 or 87·0% [82·9–90·3] *vs* 87·0% [82·9–90·3]; p=1·00 at lower thresholds), whereas stratification by age alone did not consistently improve sensitivity (76·6% [71·6–80·9] *vs* 77·2% [72·3–81·5]; p=0·48 at higher thresholds or 82·9% [78·4–86·7] *vs* 82·3% [77·7–86·1]; p=0·68 at lower thresholds; appendix p 19).

## Discussion

In this evaluation of an active case-finding programme in Uganda, the diagnostic accuracy of CAD, particularly specificity, was higher than in previous studies^11,13–15^ that restricted evaluation of CAD performance to screening participants with tuberculosis symptoms or abnormal x-rays. Under the assumption that 0·1% of people with normal or near-normal x-rays (x-ray score <0·1) have Xpert-positive tuberculosis, the AUC for CAD in detecting Xpert-positive tuberculosis in all screening participants able to provide sputum (regardless of their symptoms or x-ray results) was 0·92 (95% CI 0·90–0·94), and it would be possible for a threshold near 0·1 to meet WHO’s minimal target product profile goal^27^ of at least 90% sensitivity and at least 60% specificity for a high-sensitivity screening test. This accuracy can potentially be further improved by stratifying the CAD threshold by participant age and sex. These results speak to the utility of CAD in the population-based screening context and argue for further investigation of liberal CAD thresholds that are stratified by readily measured participant characteristics.

In this population, 5·4% of participants had an x-ray score equal to or greater than the manufacturer-recommended threshold of 0·5, and these participants had the highest probability of a positive Xpert result. However, the additional 11·6% of participants with an x-ray score between 0·1 and 0·49 still had a greater than 1% risk of having Xpert-positive tuberculosis, and excluding them from confirmatory testing limited sensitivity to 79·1%. Therefore, in the setting of community-based screening, lower CAD thresholds might be necessary to achieve the required sensitivity. Our finding of the wide radiographic spectrum of tuberculosis in communities is aligned with a recent CAD analysis of South African prevalence survey participants,^15^ in which a CAD threshold needed to be lowered to 0·18 to achieve 90% sensitivity. Because lowering the CAD threshold can strain limited resources in most areas with a high tuberculosis burden, the costs and benefits of more sensitive versus more stringent screening strategies should be weighed when setting thresholds. Formal decision analysis, informed by cost-effectiveness analysis, could help set appropriate thresholds for different populations.

We also showed that tailoring CAD thresholds according to easily identifiable individual characteristics, such as age and sex, has the potential to improve the accuracy of tuberculosis screening. Among all study participants, the prevalence of tuberculosis was four times higher in male participants than in female participants. As a result, male participants with x-ray scores of 0·52 or greater (sex-stratified threshold corresponding to 2% Xpert positivity) had a similar tuberculosis risk to female participants with x-ray scores of 0·81 or greater. In contrast, although the prevalence of tuberculosis was similar between all participants younger than 40 years and aged 40 years and older, older individuals were almost 3 times more likely to have abnormal chest imaging (ie, x-ray score ≥0·1) compared with younger individuals, likely due to a higher prevalence of other lung conditions (including unrecognised previous tuberculosis) among older adults. Consequently, among individuals who qualified for Xpert testing with abnormal chest x-ray, younger individuals were 3 times more likely to test positive on Xpert than older individuals, and individuals younger than 40 years with an x-ray score of 0·47 or greater (age-stratified threshold corresponding to 2% Xpert positivity) had a similar risk for tuberculosis to those aged 40 years and older with an x-ray score of 0·62 or more. Therefore, in settings with limited resources, the use of thresholds stratified by age and sex has the potential to increase the number of individuals with tuberculosis detected within the existing confirmatory testing capacity.

Our findings represent a promising opportunity to offer personalised screening in the context of active case-finding. In clinical settings, multiple factors, such as age, sex, HIV status, exposure history, symptoms, and chest imaging, are all incorporated into the decision-making process when deciding to test for tuberculosis. In mass-screening settings, this level of assessment is often not feasible, and simple criteria (such as the presence of tuberculosis symptoms or abnormal chest x-ray) are frequently used instead to select individuals for further testing. However, the emergence of CAD technology, which provides quantitative outputs that correlate with the probability of Xpert positivity, now make it possible to incorporate additional tuberculosis risk factors into the screening algorithm. Although the increase in sensitivity using age and sex stratification was modest (1–2%), this could nonetheless be valuable in resource-limited settings where confirmatory testing cannot be offered to all individuals who have x-ray scores above a low threshold. Future costing studies are needed to weigh these cost savings against the added cost of implementing individualised thresholds, but for simple characteristics such as age and sex, the implementation costs are expected to be minimal. If validated prospectively and in other populations, setting stratified thresholds, including lowering thresholds for individuals at high risk, should be considered.

We note that our estimates of specificity at a given sensitivity in the systematic tuberculosis screening context are high relative to some other studies, due to a difference in methodology. Even at the lowest CAD threshold for which we collected bacteriological data, 0·1, our estimate of specificity remained at 83·6% (well above the minimal target product profile goal of ≥60%). Three previous studies^13,15,18^ that evaluated qXR version 3 reported much lower specificities, ranging from under 50% to 62% for CAD thresholds providing 90% sensitivity. Those lower estimates resulted from limiting evaluations to participants with valid Xpert results, and thus to people with symptoms or abnormal chest x-ray. In contrast, by using a low CAD threshold and estimating the Xpert prevalence even below that threshold, we estimated accuracy among an entire mass-screening population, including the large segment with negative symptom screens and normal x-rays for whom tuberculosis testing is not typically performed. This inclusion of all screening-eligible individuals in accuracy estimates increases the number of true negatives (ie, no tuberculosis, with low x-ray scores) and results in higher specificities of CAD, as reported by studies^28,29^ including ours, that included individuals with normal x-rays in CAD accuracy estimation.

Our study has some limitations. First, although we used a low CAD threshold, we did not perform Xpert testing for individuals with x-ray scores less than 0·1. Because about 80% of our participants were in this category, our sensitivity estimates depend considerably on the prevalence of tuberculosis among individuals with x-ray scores less than 0·1. Our findings regarding the AUC of qXR and the added value of stratified thresholds were reasonably robust to sensitivity analysis around the prevalence of tuberculosis in this population. Moreover, we assumed a single value for the tuberculosis prevalence in these individuals with the lowest x-ray scores, but in reality it likely varies in proportion to the overall tuberculosis prevalence in each setting; future work should explore how CAD performance varies by setting. Second, we evaluated accuracy relative to a pragmatic reference standard of expectorated sputum Xpert (without sputum induction or culture). Although accuracy relative to a more comprehensive reference standard is therefore unknown, expectorated sputum Xpert confirmatory testing is common practice in systematic screening, and our estimates therefore reflect the ability of CAD to lead to detection of sputum Xpert-positive tuberculosis. Additionally, our analysis did not include pregnant participants, nor participants who did not provide expectorated sputum (9·9% of those with x-ray score ≥0·1). Although we evaluated stratification only by age, sex, or both, due to the ready availability of these variables, further work should explore stratification by other characteristics, including HIV status, which was unknown for about half of our participants, and history of tuberculosis. Third, we evaluated only one CAD software, and we did not have human readers against whom to compare CAD readings. Thus, we are unable to identify specific radiographic features associated with tuberculosis at low x-ray scores. Finally, the accuracy of stratified thresholds depended on the Xpert status of a relatively small number of people with x-ray scores between the two peaks of a bimodal x-ray score distribution and might be sensitive to changes in CAD performance. Our findings therefore warrant validation, including use of other CAD software solutions and other populations, as well as investigation into potential challenges in implementing stratified thresholds. If resources permit, evaluating tuberculosis prevalence among individuals with low X-ray scores would help further determine the utility of chest x-ray and CAD in tuberculosis screening, as CAD performance depends heavily on tuberculosis prevalence among the large number of participants with x-ray scores under the confirmatory testing threshold.

In summary, we screened over 50 000 individuals for tuberculosis in Ugandan communities using portable chest x-ray with CAD and found that, although CAD has overall high accuracy for Xpert-positive tuberculosis in this community-screening context, individuals with x-ray scores between 0·1 and 0·5 were still at elevated risk for tuberculosis. We showed that using CAD thresholds stratified by both age and sex can improve the accuracy of CAD for tuberculosis screening. Although our findings need validation, adopting lower CAD thresholds should be considered where feasible, and in settings with a high burden of tuberculosis with limited confirmatory testing capacity, stratifying thresholds by age and sex might offer a more effective and personalised approach to tuberculosis screening.

## Supplementary Material

1

## Figures and Tables

**Table 1:** Characteristics of participants who received digital chest x-ray-based tuberculosis screening with computer-aided detection software

	X-ray score <0·1 (n=43 886)	X-ray score 0·1–0·49 (n=6107)	X-ray score ≥0·5 (n=2842)	Total (n=52 835)
Median age, years	35 (25–48)	51 (37–65)	51 (37–67)	38 (26–50)

Sex				
Female	25 026 (57·0%)	3086 (50·5%)	1137 (40·0%)	29 249 (55·4%)
Male	18 860 (43·0%)	3021 (49·5%)	1705 (60·0%)	23 586 (44·6%)

HIV status*				
Known positive	2726 (6·2%)	457 (7·5%)	295 (10·4%)	3478 (6·6%)
Negative, with a test within the past year	21 776 (49·6%)	2704 (44·3%)	1189 (41·8%)	25 669 (48·6%)
Unknown†	19 384 (44·2%)	2946 (48·2%)	1358 (47·8%)	23 688 (44·8%)

Previous tuberculosis treatment	257 (0·6%)	123 (2·0%)	344 (12·1%)	724 (1·4%)

Previous household tuberculosis exposure	595 (1·4%)	104 (1·7%)	54 (1·9%)	753 (1·4%)

Current smoking‡	410/12 081 (3·4%)	114/1501 (7·6%)	79/753 (10·5%)	603/14 335 (4·2%)

Symptoms within 30 days of enrolment				
Any cough	12 945 (29·5%)	2299 (37·6%)	1613 (56·8%)	16 857 (31·9%)
Cough ≥2 weeks	6681 (15·2%)	1435 (23·5%)	1227 (43·2%)	9343 (17·7%)
Fever	8105 (18·5%)	1320 (21·6%)	728 (25·6%)	10 153 (19·2%)
Night sweats	1439 (3·3%)	294 (4·8%)	236 (8·3%)	1969 (3·7%)
Weight loss	909 (2·1%)	193 (3·2%)	181 (6·4%)	1283 (2·4%)
Any tuberculosis symptom§	17 876 (40·7%)	2963 (48·5%)	1865 (65·6%)	22 704 (43·0%)

Data are median (IQR), n (%), or n/N (%).

* Based on self-reporting.

† Includes 23 participants who declined to disclose HIV status.

‡ Only asked for participants enrolled after Nov 11, 2023.

§ Any cough, fever, night sweats, or weight loss.

**Table 2:** Proportion of participants with positive* sputum Xpert MTB/RIF Ultra results among those with valid results, by x-ray score

	Male (n=3843)	Female (n=3376)	Aged <40 years (n=1999)	Aged ≥40 years (n=5220)	All tested participants (n=7219)
0·1–0·19	14/1042 (1·3%)	3/1124 (0·3%)	7/671 (1·0%)	10/1495 (0·7%)	17/2166 (0·8%)
0·2–0·29	8/590 (1·4%)	3/586 (0·5%)	4/299 (1·3%)	7/877 (0·8%)	11/1176 (0·9%)
0·3–0·39	9/367 (2·5%)	7/380 (1·8%)	9/173 (5·2%)	7/574 (1·2%)	16/747 (2·1%)
0·4–0·49	8/250 (3·2%)	3/248 (1·2%)	3/106 (2·8%)	8/392 (2·0%)	11/498 (2·2%)
0·5–0·59	9/240 (3·8%)	3/181 (1·7%)	3/87 (3·4%)	9/334 (2·7%)	12/421 (2·9%)
0·6–0·69	9/198 (4·5%)	3/163 (1·8%)	4/75 (5·3%)	8/286 (2·8%)	12/361 (3·3%)
0·7–0·79	9/168 (5·4%)	1/156 (0·6%)	7/89 (7·9%)	3/235 (1·3%)	10/324 (3·1%)
0·8–0·89	14/198 (7·1%)	7/178 (3·9%)	6/97 (6·2%)	15/279 (5·4%)	21/376 (5·6%)
0·9–1·0	209/789 (26·5%)	63/359 (17·5%)	150/402 (37·3%)	122/746 (16·4%)	272/1148 (23·7%)
Any x-ray score ≥0·1	289/3843 (7·5%)	93/3376 (2·8%)	193/1999 (9·7%)	189/5220 (3·6%)	382/7219 (5·3%)

* Including trace-positive.

**Table 3:** Sensitivity and specificity of qXR thresholds stratified by both age and sex for detecting Xpert MTB/RIF Ultra-positive tuberculosis, compared with universal thresholds of matching specificity

	Fixed-specificity comparison, higher sensitivities		Fixed-specificity comparison, lower sensitivities	
	Thresholds stratified by both age and sex*	Universal threshold†	Thresholds stratified by both age and sex‡	Universal threshold§
**Sensitivity estimates**				
Assumed prevalence 0·05%	89·7% (85·7–92·6)	88·0% (83·8–91·2)	81·0% (76·2–85·0)	79·0% (74·0–83·2)
Assumed prevalence 0·1%	85·1% (80·8–88·6)	83·5% (79·1–87·2)	76·9% (71·9–81·2)	75·0% (69·9–79·5)
Assumed prevalence 0·2%	77·3% (72·6–81·4)	75·9% (71·1–80·1)	69·8% (64·8–74·4)	68·1% (63·0–72·8)
Assumed prevalence 0·3%	70·6% (65·8–75·0)	69·3% (64·5–73·7)	63·8% (58·8–68·4)	62·2% (57·2–66·9)

**Specificity estimates**				
Assumed prevalence 0·05–0·3%¶	91·0% (90·7–91·3)	91·0% (90·7–91·3)	96·1% (95·9–96·3)	96·1% (95·9–96·3)

Data are % (95% CI). Assumed prevalences are the assumed tuberculosis prevalence when x-ray scores are <0·1. qXR=computer-aided detection software qXR version 3.2.

* Correspond to the estimated scores at which approximately 1% of participants in each subgroup would test positive. Threshold ≥0·11 for those aged 15–39 years and male; threshold ≥0·25 for those aged ≥40 years and male; threshold ≥0·28 for those aged 15–39 years and female; and threshold 0·44 for those aged ≥40 years and female.

† Chosen to match the 91·0% aggregate specificity of thresholds stratified by both age and sex. Threshold ≥0·26 for all.

‡ Correspond to the estimated scores at which approximately 2% of participants in each subgroup would test positive. Threshold ≥0·47 for those aged 15–39 years and male; threshold ≥0·53 for those aged ≥40 years and male; threshold ≥0·52 for those aged 15–39 years and female; threshold 0·89 for those aged ≥40 years and female.

§ Chosen to match the 96·1% aggregate specificity of thresholds stratified by both age and sex. Threshold ≥0·65 for all.

¶ Specificities remained the same when tuberculosis prevalence among individuals with x-ray scores <0·1 was assumed to be 0·05%, 0·1%, 0·2%, or 0·3%.

## Data Availability

The de-identified dataset of participant demographics, x-ray scores, and Xpert results used for this study and a data dictionary will be available on reasonable request. Data sharing will be limited to non-commercial research use only. Requests should include a proposal outlining the intended use and methodology and will be subject to review and approval. Requests can be directed to ekendall@jhmi.edu.

## Research in context

### Evidence before this study

WHO has endorsed computer-aided detection (CAD) as a screening tool for tuberculosis, but the appropriate x-ray score that triggers further diagnostic evaluation for tuberculosis (the CAD threshold) varies by population. WHO recommends determining the appropriate CAD threshold for specific settings and considering unique thresholds for specific populations, including older age groups, among whom CAD might perform poorly. We searched PubMed for articles published from database inception to Sept 9, 2024, using the search terms “tuberculosis” AND (“computer-aided detection” OR “computer aided detection” OR “CAD” OR “computer-aided reading” OR “computer aided reading” OR “artificial intelligence”), and found 704 articles. Among them, we identified articles published in English that evaluated the performance of CAD for tuberculosis screening and additionally reviewed relevant references. Most previous studies reported areas under the curve (AUCs) ranging from 0·76 to 0·88 but limited their evaluations to individuals with symptoms or abnormal chest x-rays. Some previous studies identified subgroups (including older individuals and people with previous tuberculosis) among whom CAD had lower-than-average AUCs, and authors discussed how the prevalence of such characteristics could affect the optimal value of a population-wide CAD threshold; however, none estimated the accuracy that could be gained with adjusting CAD thresholds between individuals based on personal characteristics.

### Added value of this study

In this study, consenting adolescents and adults in a high-prevalence setting were offered chest x-ray screening regardless of symptoms. A very low x-ray score cutoff (CAD software qXR version 3.2 tuberculosis score of 0·1 on a 0–1 scale) was used to select individuals for confirmatory sputum molecular testing, enabling the detection of radiographically mild forms of tuberculosis and facilitating comparisons of diagnostic accuracy at different CAD thresholds. To assess CAD performance among all x-ray screening participants capable of providing sputum, a tuberculosis prevalence of 0·1% was assumed for individuals with x-ray scores less than 0·1 who were not offered sputum testing. Using this symptom-neutral evaluation of CAD with expansive criteria for bacteriological testing allowed us to account for all individuals screened when estimating the accuracy of CAD, and our AUC estimate of 0·92 suggests that accuracy might be higher than previously reported—although estimates depend on the prevalence of tuberculosis among people with x-ray scores less than 0·1. We also found that a very low CAD score threshold (less than 0·1 for qXR version 3.2) is needed to meet WHO’s minimal target product profile goal of at least 90% sensitivity and at least 60% specificity for a high-sensitivity screening test. Our findings also suggest that adjusting CAD thresholds to align positive predictive values between demographic groups could achieve small gains in the sensitivity of x-ray screening (a 1–2% increase in sensitivity after adjusting CAD thresholds by age and sex strata) without affecting specificity.

### Implications of all the available evidence

To obtain high sensitivity with CAD screening in high-prevalence settings, low score thresholds might be needed. However, countries with a high burden of tuberculosis often do not have sufficient resources to test all individuals above a low threshold. In such settings, adjusting CAD thresholds based on individual characteristics associated with tuberculosis prevalence (eg, male sex) and those associated with false-positive x-ray results (eg, older age) can potentially improve the efficiency of tuberculosis screening programmes.

## References

[1] WHO. Global Tuberculosis Report 2023. World Health Organization, 2023.

[2] Burke RM, Nliwasa M, Feasey HRA, Community-based active case-finding interventions for tuberculosis: a systematic review. Lancet Public Health 2021; 6: e283–99. 33765456 10.1016/S2468-2667(21)00033-5 PMC8082281

[3] WHO. WHO consolidated guidelines on tuberculosis: Module 2: screening–systematic screening for tuberculosis disease. World Health Organization, 2021. 33822560

[4] WHO. Determining the local calibration of computer-assisted detection (CAD) thresholds and other parameters: a toolkit to support the effective use of CAD for TB screening. World Health Organization, 2021.

[5] Qin ZZ, Sander MS, Rai B, Using artificial intelligence to read chest radiographs for tuberculosis detection: a multi-site evaluation of the diagnostic accuracy of three deep learning systems. Sci Rep 2019; 9: 15000. 31628424 10.1038/s41598-019-51503-3 PMC6802077

[6] Breuninger M, van Ginneken B, Philipsen RH, Diagnostic accuracy of computer-aided detection of pulmonary tuberculosis in chest radiographs: a validation study from sub-Saharan Africa. PLoS One 2014; 9: e106381. 25192172 10.1371/journal.pone.0106381 PMC4156349

[7] Khan FA, Majidulla A, Tavaziva G, Chest x-ray analysis with deep learning-based software as a triage test for pulmonary tuberculosis: a prospective study of diagnostic accuracy for culture-confirmed disease. Lancet Digit Health 2020; 2: e573–81. 33328086 10.1016/S2589-7500(20)30221-1

[8] Melendez J, Sánchez CI, Philipsen RH, An automated tuberculosis screening strategy combining x-ray-based computer-aided detection and clinical information. Sci Rep 2016; 6: 25265. 27126741 10.1038/srep25265 PMC4850474

[9] Muyoyeta M, Maduskar P, Moyo M, The sensitivity and specificity of using a computer aided diagnosis program for automatically scoring chest x-rays of presumptive TB patients compared with Xpert MTB/RIF in Lusaka Zambia. PLoS One 2014; 9: e93757. 24705629 10.1371/journal.pone.0093757 PMC3976315

[10] Qin ZZ, Ahmed S, Sarker MS, Tuberculosis detection from chest x-rays for triaging in a high tuberculosis-burden setting: an evaluation of five artificial intelligence algorithms. Lancet Digit Health 2021; 3: e543–54. 34446265 10.1016/S2589-7500(21)00116-3

[11] Fehr J, Gunda R, Siedner MJ, CAD4TB software updates: different triaging thresholds require caution by users and regulation by authorities. Int J Tuberc Lung Dis 2023; 27: 157–60. 36853104 10.5588/ijtld.22.0437 PMC9904401

[12] Kik SV, Gelaw SM, Ruhwald M, Diagnostic accuracy of chest x-ray interpretation for tuberculosis by three artificial intelligence-based software in a screening use-case: an individual patient meta-analysis of global data. medRxiv 2022; published online Jan 27. 10.1101/2022.01.24.22269730 (preprint).

[13] Codlin AJ, Dao TP, Vo LNQ, Independent evaluation of 12 artificial intelligence solutions for the detection of tuberculosis. Sci Rep 2021; 11: 23895. 34903808 10.1038/s41598-021-03265-0 PMC8668935

[14] Scott AJ, Perumal T, Hohlfeld A, Diagnostic accuracy of computer-aided detection during active case finding for pulmonary tuberculosis in Africa: a systematic review and meta-analysis. Open Forum Infect Dis 2024; 11: ofae020. 38328498 10.1093/ofid/ofae020 PMC10849117

[15] Qin ZZ, Van der Walt M, Moyo S, Computer-aided detection of tuberculosis from chest radiographs in a tuberculosis prevalence survey in South Africa: external validation and modelled impacts of commercially available artificial intelligence software. Lancet Digit Health 2024; 6: e605–13. 39033067 10.1016/S2589-7500(24)00118-3 PMC11339183

[16] Nguyen HV, Tiemersma E, Nguyen NV, Nguyen HB, Cobelens F. Disease transmission by patients with subclinical tuberculosis. Clin Infect Dis 2023; 76: 2000–06. 36660850 10.1093/cid/ciad027 PMC10249982

[17] MacPherson P, Webb EL, Kamchedzera W, Computer-aided x-ray screening for tuberculosis and HIV testing among adults with cough in Malawi (the PROSPECT study): a randomised trial and cost-effectiveness analysis. PLoS Med 2021; 18: e1003752. 34499665 10.1371/journal.pmed.1003752 PMC8459969

[18] Kagujje M, Kerkhoff AD, Nteeni M, Dunn I, Mateyo K, Muyoyeta M. The performance of computer-aided detection digital chest x-ray reading technologies for triage of active tuberculosis among persons with a history of previous tuberculosis. Clin Infect Dis 2023; 76 e894–901. 36004409 10.1093/cid/ciac679 PMC9907528

[19] Fehr J, Konigorski S, Olivier S, , the Vukuzazi Team. Computer-aided interpretation of chest radiography reveals the spectrum of tuberculosis in rural South Africa. NPJ Digit Med 2021; 4: 106. 34215836 10.1038/s41746-021-00471-y PMC8253848

[20] Moodley N, Velen K, Saimen A, Zakhura N, Churchyard G, Charalambous S. Digital chest radiography enhances screening efficiency for pulmonary tuberculosis in primary health clinics in South Africa. Clin Infect Dis 2022; 74: 1650–58. 34313729 10.1093/cid/ciab644

[21] Horton KC, MacPherson P, Houben RM, White RG, Corbett EL. Sex differences in tuberculosis burden and notifications in low- and middle-income countries: a systematic review and meta-analysis. PLoS Med 2016; 13: e1002119. 27598345 10.1371/journal.pmed.1002119 PMC5012571

[22] Ledesma JR, Ma J, Vongpradith A, Global, regional, and national sex differences in the global burden of tuberculosis by HIV status, 1990–2019: results from the Global Burden of Disease Study 2019. Lancet Infect Dis 2022; 22: 222–41. 34563275 10.1016/S1473-3099(21)00449-7 PMC8799634

[23] Mungai BN, Joekes E, Masini E, ‘If not TB, what could it be?’ Chest X-ray findings from the 2016 Kenya Tuberculosis Prevalence Survey. Thorax 2021; 76: 607–14. 33504563 10.1136/thoraxjnl-2020-216123 PMC8223623

[24] The Republic of Uganda Ministry of Health. The Uganda national tuberculosis prevalence survey, 2014–2015 survey report. Ministry of Health, 2016.

[25] Pya N, Wood SN. Shape constrained additive models. Stat Comput 2015; 25: 543–59.

[26] SungJ, NantaleM, NalutaayaA, Long-term risk of tuberculosis among individuals with Xpert Ultra trace screening results in Uganda: a longitudinal follow-up study. Lancet Infect Dis 2025; published online Oct 7. 10.1016/S1473-3099(25) 00536–5.41072452

[27] WHO. Target product profiles for tuberculosis screening tests. World Health Organization, 2025.

[28] Keter AK, Vanobberghen F, Lynen L, Simultaneous alleviation of verification and reference standard biases in a community-based tuberculosis screening study using Bayesian latent class analysis. PLoS One 2024; 19: e0305126. 38857227 10.1371/journal.pone.0305126 PMC11164341

[29] Mungai B, Ong’angò J, Ku CC, Accuracy of computer-aided chest x-ray in community-based tuberculosis screening: lessons from the 2016 Kenya National Tuberculosis Prevalence Survey. PLOS Glob Public Health 2022; 2: e0001272. 36962655 10.1371/journal.pgph.0001272 PMC10022380

